# Elucidating the contribution of wild related species on autochthonous pear germplasm: A case study from Mount Etna

**DOI:** 10.1371/journal.pone.0198512

**Published:** 2018-06-01

**Authors:** Stefania Bennici, Giuseppina Las Casas, Gaetano Distefano, Mario Di Guardo, Alberto Continella, Filippo Ferlito, Alessandra Gentile, Stefano La Malfa

**Affiliations:** 1 Dipartimento di Agricoltura, Alimentazione e Ambiente, University of Catania, Catania, Italy; 2 Consiglio per la ricerca in agricoltura e l’analisi dell’economia agraria, Centro di Ricerca Olivicoltura, Frutticoltura e Agrumicoltura (CREA-OFA), Acireale, Italy; Instituto Agricultura Sostenible, SPAIN

## Abstract

The pear (genus *Pyrus*) is one of the most ancient and widely cultivated tree fruit crops in temperate climates. The Mount Etna area claims a large number of pear varieties differentiated due to a long history of cultivation and environmental variability, making this area particularly suitable for genetic studies. Ninety-five pear individuals were genotyped using the simple sequence repeat (SSR) methodology interrogating both the nuclear (nDNA) and chloroplast DNA (cpDNA) to combine an investigation of maternal inheritance of chloroplast SSRs (cpSSRs) with the high informativity of nuclear SSRs (nSSRs). The germplasm was selected ad hoc to include wild genotypes, local varieties, and national and international cultivated varieties. The objectives of this study were as follows: (i) estimate the level of differentiation within local varieties; (ii) elucidate the phylogenetic relationships between the cultivated genotypes and wild accessions; and (iii) estimate the potential genetic flow and the relationship among the germplasms in our analysis. Eight nSSRs detected a total of 136 alleles with an average minor allelic frequency and observed heterozygosity of 0.29 and 0.65, respectively, whereas cpSSRs allowed identification of eight haplotypes (S4 Table). These results shed light on the genetic relatedness between Italian varieties and wild genotypes. Among the wild species, compared with *P*. *amygdaliformis*, few *P*. *pyraster* genotypes exhibited higher genetic similarity to local pear varieties. Our analysis revealed the presence of genetic stratification with a ‘wild’ subpopulation characterizing the genetic makeup of wild species and the international cultivated varieties exhibiting the predominance of the ‘cultivated’ subpopulation.

## Introduction

The pear (*Pyrus* spp.) is one of the most cultivated fruit crops in temperate zones. *Pyrus* species are traditionally divided into two groups based on domestication area and geographic distribution. European pears (*P*. *communis*) are cultivated mainly in Europe and the U.S., and Asian pears (*P*. *pyrifolia*, *P*. *bretschneideri* and *P*. *ussuriensis*) grow in East Asian countries. The genus *Pyrus* belongs to the family *Rosaceae*, subtribe *Pyrinae* and contains at least 22 widely recognized primary species, all indigenous to Asia, Europe, and the mountainous area of North America. The worldwide production of European pear relies on a few main cultivars released from the late 18^th^ century onward (or derived from those cultivars).

Although Sicily is not important in pear production and does not include large areas of production, it exhibits significant germplasm diversity. In the Mount Etna area, the large amount of pear biodiversity could be related to ancient practises of cultivation and seed propagation combined with the variety of favourable microclimates, soils and orographic conditions. In addition, pear cultivation in Sicily was historically characterized by a wide use of wild pears (*P*. *amygdaliformis* Vill.; *P*. *communis* ssp. *pyraster* L.) as rootstocks, an agronomical practise that increases the hardiness and longevity of the trees [1]. The most common species of wild pear (*P*. *pyraster* = *P*. *communis* ssp. *pyraster* L.) is a woody plant closely related to the European pear. This species comes from the western Black Sea region. Its distribution extends from the British Isles to Latvia [2] and is widespread in Sicily.

The local pear germplasm of Mount Etna could represent an important source of ecological interest for specific characteristics, such as high drought resistance, low chill unit requirement, adaptation to hot and dry summer conditions and low pest and disease incidence, all of which are pivotal characteristics to consider in the establishment of novel breeding programmes. As such, ascertaining the genetic relationships and phylogeny of the genus *Pyrus* and the potential contribution of wild related species to its origin would be advisable, given the promising results obtained in other Rosaceae tree crops, such as apple, peach and cherry [3–5].

DNA markers have become powerful tools for cultivar identification, evaluation of genetic diversity and parentage analysis. Studies on the genetic relationships of pear genotypes were performed using nuclear [6] and chloroplast restriction fragment length polymorphisms (RFLPs) [7], random amplification of polymorphic DNA (RAPD) [8–13], amplification fragment length polymorphisms (AFLPs) [14] and inter-simple sequence repeats (ISSR) [15]. In addition to these markers, SSRs, in particular, have been widely used for genetic relationship and diversity studies given their hyper-variability, co-dominant nature, prevalent locus-specificity, and random genome-wide distributions. A large set of SSR markers has been developed from Japanese and European pear [16–18] and subsequently used for genetic characterization and identification of different *Pyrus* species [19–22]. In addition, SSR markers present a high transferability between related species and genera. SSR markers developed in apple (*Malus* x *domestica* Borkh.) present a high level of transferability and polymorphisms in the subtribe *Pyrinae* (formerly the *Maloideae*), allowing their use to assess genetic diversity and cultivar identification in pear [23–26]. Markers for several intergenic spacer and intron regions in the chloroplast genome have been applied for phylogenetic analyses in *Pyrus* [27–30]. The maternally inherited chloroplast genome is much smaller than the nuclear genome and is more conserved [31–32]. Thus, this genome could be used effectively to determine the parentage germplasms of hybrids. In addition, cytoplasmic markers appear to be suitable for overcoming the multiple gene copy problem in polyploid phylogenetics, as occasionally reported in pear [33]. These markers have been widely used in angiosperms for genetic diversity and phylogenetic relationship studies to investigate the evolution of plants and gene flow in natural populations [34]. Nuclear and cytoplasmic DNA markers have been employed in *Pyrus* for both parentage and taxonomy studies [7, 35–36]. Genetic diversity, structure and hybridization rates were evaluated in several collections of wild *P*. *pyraster*, *P*. *communis*, *P*. *pyrifolia* and *P*. *ussuriensis* using nuclear and chloroplast SSR markers for improving preservation measures [37–40]. The use of molecular markers could greatly improve the cost-effectiveness of pear breeding. The traditional breeding of pears is a costly and time-consuming process due to the long juvenile period and large size of the plants (requiring great time and space investments) as well as the genetic complexity of *Pyrus* resulting from the self-incompatibility of the genus. The use of molecular markers could have a direct positive implication for the genetic characterization of the germplasm collection, laying a foundation for use of genetic polymorphisms to make predictions of phenotype changes through marker-trait association analysis.

In the present study, nuclear (nSSR) and chloroplast (cpSSR) microsatellites were used (i) to estimate the level of differentiation within the cultivated genotypes and the wild accessions; (ii) to elucidate phylogenetic relationships between the cultivated genotypes and the wild accessions; and (iii) to estimate the potential genetic flow between and the relationship among local Sicilian pear genotypes, native wild species and international varieties.

## Materials and methods

### Plant material and DNA extraction

**Fig 1:**
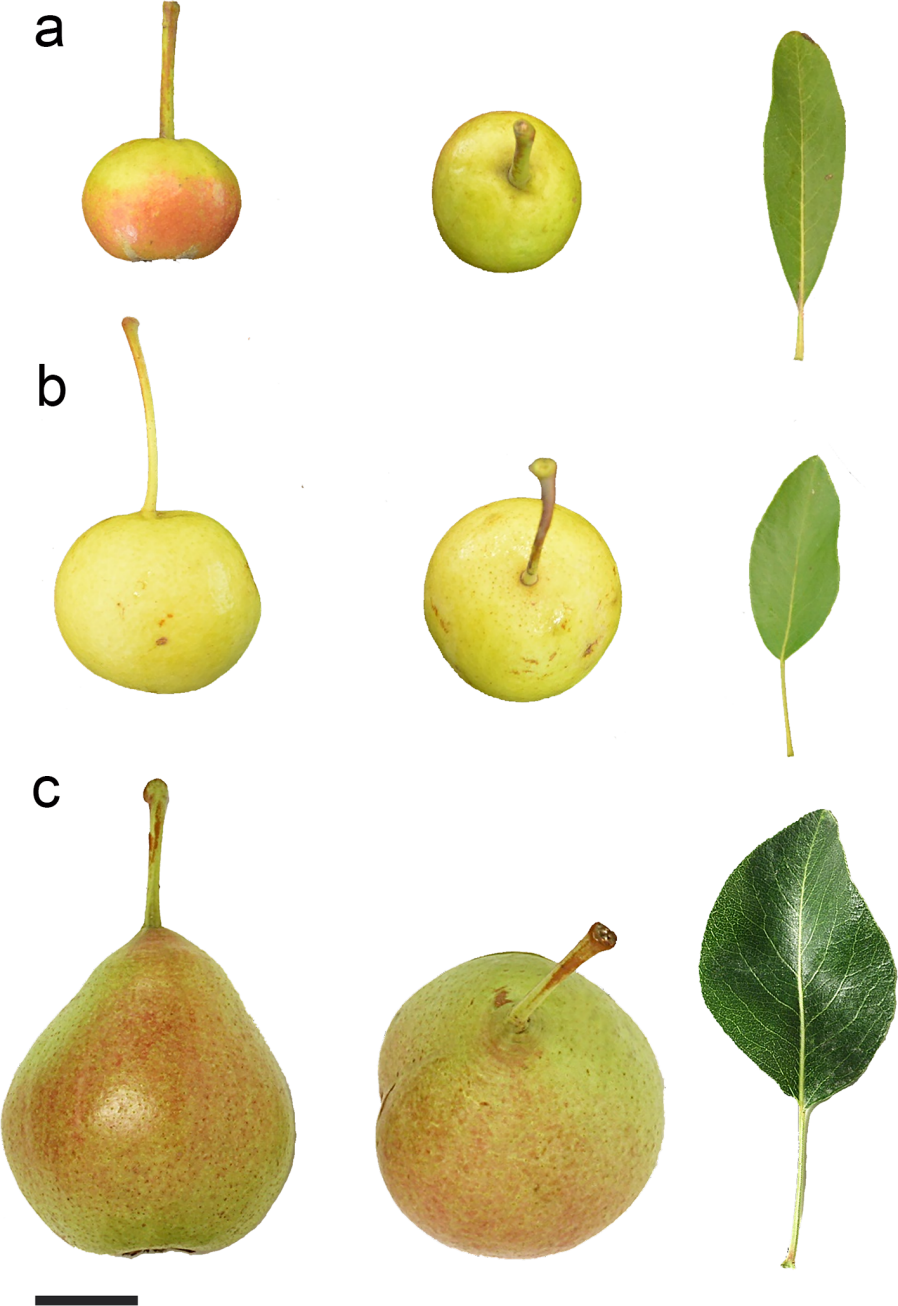
Comparison of pear germplasm. Section of fruit and leaves of *P*. *amygdaliformis* (a), *P*. *pyraster* (b) and widespread cultivated traditional variety (‘Spineddu’; c) on Mount Etna (scale bar = 2 cm).

Ninety-five pear genotypes were used in this study (Table 1), including 46 local varieties (LV) and 21 wild related species (RS) collected from Etna district (Italy), 19 nationally cultivated varieties (NCV) and 9 internationally cultivated varieties (ICV) (Fig 1). Genotypes were sampled from different sites as specified in S1 Table.

**Table 1:** List of genotypes used in this study.

Species	Accession name	Status	Origin	PC1	PC2	N-J Cluster
*P*. *communis*	Adamo	LV	Italy (Sicily)	-0.375	0.884	A
*P*. *communis*	Alessio	LV	Italy (Sicily)	3.522	-2.866	A
*P*. *communis*	Angelico	NCV	Italy (Sicily)	-	-	-
*P*. *communis*	Angelico Doppio	LV	Italy (Sicily)	-1.074	-0.303	B1
*P*. *communis*	Azzone di Cassone	LV	Italy (Sicily)	2.346	0.065	B1
*P*. *communis*	Bella di Giugno	NCV	Italy (Sicily)	1.638	-1.36	A
*P*. *communis*	Bergamotto	NCV	Italy (Sicily)	1.863	-0.451	B1
*P*. *communis*	Bianchetto (1)	NCV	Italy (Sicily)	-2.328	3.015	B1
*P*. *communis*	Bianchetto (2)	NCV	Italy (Sicily)	-	-	B1
*P*. *communis*	Bianchettone	LV	Italy (Sicily)	-0.953	0.787	A
*P*. *communis*	Bruttu Beddu	LV	Italy (Sicily)	2.079	-2.636	B1
*P*. *communis*	Buona Luisa	NCV	Italy (Sicily)	4.181	-0.809	A
*P*. *communis*	Butirra	NCV	Italy (Sicily)	4.037	-0.959	A
*P*. *communis*	Campana	NCV	Italy (Sicily)	-	-	B1
*P*. *communis*	Catanese	LV	Italy (Sicily)	-0.765	2.954	B1
*P*. *communis*	Cavaliere	LV	Italy (Sicily)	-	-	B1
*P*. *communis*	Chiuzzu	LV	Italy (Sicily)	-0.668	-0.629	C
*P*. *communis*	Coscia	NCV	Italy (Sicily)	3.708	-0.876	A
*P*. *communis*	Duchessa D'angio'	LV	Italy (Sicily)	4.228	-0.799	A
*P*. *communis*	Faccia Donna	LV	Italy (Sicily)	-2.808	4.265	B1
*P*. *communis*	Faccibedda	LV	Italy (Sicily)	-0.411	2.388	B2
*P*. *communis*	Franconello	LV	Italy (Sicily)	-	-	B2
*P*. *communis*	Garibaldi	LV	Italy (Sicily)	-	-	B1
*P*. *communis*	Garofalo	NCV	Italy (Sicily)	-3.246	4.783	B1
*P*. *communis*	Gentile	NCV	Italy (Sicily)	1.985	-1.724	A
*P*. *communis*	Ialufaru	LV	Italy (Sicily)	-2.887	4.961	B1
*P*. *communis*	Ianculiddu	LV	Italy (Sicily)	-2.028	1.562	B1
*P*. *communis*	Iazzuleddu	LV	Italy (Sicily)	1.984	-0.044	B1
*P*. *communis*	Mezza Campana	LV	Italy (Sicily)	-	-	B1
*P*. *communis*	Moscatello (2)	NCV	Italy (Sicily)	-1.621	1.701	C
*P*. *communis*	Moscatello (1)	NCV	Italy (Sicily)	-1.59	0.75	B1
*P*. *communis*	Moscatello Maiolino	LV	Italy (Sicily)	-	-	B2
*P*. *communis*	Moscatello Nero	LV	Italy (Sicily)	-1.578	1.749	B1
*P*. *communis*	Paradiso/Confittaru	LV	Italy (Sicily)	-0.095	2.018	B2
*P*. *communis*	Pasqualino	LV	Italy (Sicily)	1.448	-0.842	B1
*P*. *communis*	Pauluzzo	LV	Italy (Sicily)	-0.411	2.388	B2
*P*. *communis*	Pergolesi	LV	Italy (Sicily)	3.268	-0.896	A
*P*. *communis*	Pero Angelico	LV	Italy (Sicily)	3.669	-1.026	A
*P*. *communis*	Piccola Dolce	LV	Italy (Sicily)	-	-	B1
*P*. *communis*	Piridda	LV	Italy (Sicily)	-2.247	1.716	B1
*P*. *communis*	Piru Mulinciana	LV	Italy (Sicily)	4.659	-0.3	A
*P*. *communis*	Piru Pizzu	LV	Italy (Sicily)	-0.389	-0.105	B1
*P*. *communis*	Pisciazzaru	LV	Italy (Sicily)	-0.093	1.427	A
*P*. *communis*	Pistacchino	LV	Italy (Sicily)	0.342	-0.388	B1
*P*. *communis*	Putiru d'Estate	LV	Italy (Sicily)	1.406	-0.602	A
*P*. *communis*	Putiru d'Inverno	LV	Italy (Sicily)	2.694	-1.221	B1
*P*. *communis*	Razzuolo Rosata	LV	Italy (Sicily)	-1.398	1.35	A
*P*. *communis*	Regina	LV	Italy (Sicily)	-	-	B2
*P*. *communis*	Rosa	LV	Italy (Sicily)	-1.464	1.289	B2
*P*. *communis*	San Cono	LV	Italy (Sicily)	0.615	-0.105	A
*P*. *communis*	San Giovanni	NCV	Italy (Sicily)	-2.338	2.525	B1
*P*. *communis*	San Giovannino	LV	Italy (Sicily)	-2.702	4.295	B1
*P*. *communis*	San Pietro	NCV	Italy (Sicily)	-0.544	2.702	B2
*P*. *communis*	Santa Caterina	LV	Italy (Sicily)	-1.794	1.875	B2
*P*. *communis*	Savino	LV	Italy (Sicily)	-	-	-
*P*. *communis*	Sciaduna	LV	Italy (Sicily)	-0.811	0.762	B3
*P*. *communis*	Spadona	NCV	Italy (Sicily)	-2.048	2.645	B1
*P*. *communis*	Spineddu	NCV	Italy (Sicily)	-0.529	1.14	B3
*P*. *communis*	Tabaccaro	LV	Italy (Sicily)	0.082	1.734	A
*P*. *communis*	Ucciarduni	NCV	Italy (Sicily)	-	-	B1
*P*. *communis*	Urzi'	LV	Italy (Sicily)	-	-	A
*P*. *communis*	Villalba	LV	Italy (Sicily)	-	-	A
*P*. *communis*	Virgolese	NCV	Italy (Sicily)	3.268	-0.896	A
*P*. *communis*	Zio Pietro	LV	Italy (Sicily)	3.723	-0.469	A
*P*. *communis*	Zuccareddu	LV	Italy (Sicily)	-0.017	1.89	B1
*P*. *amygdaliformis*	1	RS	Italy (Sicily)	-4.935	-7.597	B3
*P*. *amygdaliformis*	2	RS	Italy (Sicily)	-4.603	-7.797	B3
*P*. *amygdaliformis*	3	RS	Italy (Sicily)	-3.673	0.183	B3
*P*. *amygdaliformis*	4	RS	Italy (Sicily)	-4.977	-2.986	B3
*P*. *amygdaliformis*	5	RS	Italy (Sicily)	-	-	B3
*P*. *amygdaliformis*	6	RS	Italy (Sicily)	-	-	B3
*P*. *amygdaliformis*	7	RS	Italy (Sicily)	-6.165	-6.905	B3
*P*. *amygdaliformis*	8	RS	Italy (Sicily)	-3.517	-4.833	B3
*P*. *amygdaliformis*	9	RS	Italy (Sicily)	-4.636	-7.798	B3
*P*. *amygdaliformis*	10	RS	Italy (Sicily)	-	-	-
*P*. *pyraster*	1	RS	Italy (Sicily)	-2.307	-1.648	B1
*P*. *pyraster*	2	RS	Italy (Sicily)	-	-	A
*P*. *pyraster*	3	RS	Italy (Sicily)	-0.238	0.719	A
*P*. *pyraster*	4	RS	Italy (Sicily)	-	-	B3
*P*. *pyraster*	5	RS	Italy (Sicily)	-	-	B3
*P*. *pyraster*	6	RS	Italy (Sicily)	-	-	-
*P*. *pyraster*	7	RS	Italy (Sicily)	-0.487	-1.221	B3
*P*. *pyraster*	8	RS	Italy (Sicily)	-3.946	2.234	B3
*P*. *pyraster*	9	RS	Italy (Sicily)	-2.819	2.042	A
*P*. *pyraster*	10	RS	Italy (Sicily)	2.606	-1.052	A
*P*. *pyraster*	11	RS	Italy (Sicily)	-1.293	-0.839	B1
*P*. *communis*	Abate Fetel	ICV	France	3.164	-1.187	B1
*P*. *communis*	Butirra Hardy	ICV	France	0.722	1.152	B1
*P*. *communis*	Decana del Comizio	ICV	France	1.646	0.616	A
*P*. *communis*	Dr. Jules Guyot	ICV	France	-	-	A
*P*. *communis*	Kaiser	ICV	France	2.066	-0.38	B1
*P*. *communis*	Max Red Bartlett	ICV	USA	6.106	-1.137	A
*P*. *communis*	Old Home	ICV	USA	-	-	A
*P*. *communis*	Harrow Sweet	ICV	Canada	3.592	-0.126	A
*P*. *communis*	William’s	ICV	England	6.106	-1.137	A

Plants were divided into four groups: wild Related Species (RS), Local Varieties (LV), National Commercial Varieties (NCV), International Commercial Varieties (ICV). Coordinates on the principal component analysis (PC1 and PC2) and assigned cluster on the neighbour-joining tree (NJ Cluster) are also reported.

Genomic DNA was extracted from fresh leaves using ISOLATE II Plant DNA Kits (Bioline, Meridian Life Science, Memphis, TN, USA). The quantities and qualities of the extracted DNA samples were determined using a Nanodrop 2000 (Thermo Scientific, Waltham, MA, USA) spectrophotometer and agarose gel electrophoresis. DNA samples were stored at -20°C.

### SSR analysis by capillary electrophoresis

PCR amplification was performed using four chloroplast SSR primer pairs derived from the pear genome and eight nuclear SSR primer pairs derived from the pear and apple genomes (Table 2). PCR reactions were each performed in a 15-μl volume containing 40 ng genomic DNA, 1x PCR buffer II, 2 mM magnesium chloride, 0.2 mM dNTPs, 0.3 μM each primer, 0.13 μM 5’-fluorescently labelled M13F primer (CAC GAC GTT GTA AAA CGA C) tagged with 6-FAM, NED, VIC or PET and 1U of MyTaq DNA polymerase (Bioline). Amplifications were conducted using a programme with an initial denaturation step at 95°C for 15 min followed by 35 cycles at 95°C for 30 sec, 52–55°C for 30 sec and 72°C for 45 min with a final cycle of 72°C for 15 min. A 0.4- to 0.6-μl aliquot of PCR product (depending on the performance of amplification of each primer pair) was mixed with 13 μl of formamide and 0.3 μl of LIZ-500 size standard and denatured at 95°C for 5 min. Up to four PCR products labelled with 6-FAM, PET, VIC or NED were pooled before separation in the ABI 3130 Genetic Analyser (Applied Biosystems, Foster City, CA, USA) and subjected to subsequent analysis using GeneMapper 4.0 software.

**Table 2:** SSR markers used in this study.

Marker	Reference	Repeat type	Annealing temperature	Origin
NH013a	Wuyun *et al*. (2015)	(T)_11_	52	Pear
CH02h11a	Wuyun *et al*. (2015)	(T)_11_	55	Pear
BGT23b	Wuyun *et al*. (2015)	(T)_10_	55	Pear
CH05d04	Wuyun *et al*. (2015)	(A)_19_	52	Pear
TsuENH025	Yamamoto *et al*. (2002b)	(AG)_13_	52	Pear
TsuENH026	Liebhard *et al*. (2002)	PERF	52	Apple
NH015a	Yamamoto *et al*. (2002a)	(TC)_18_._5_	52	Pear
CH04e03	Liebhard *et al*. (2002)	COMP	52	Apple
PCHSSR3	Nishitani *et al*. (2009)	(CT)_10_._5_	55	Pear
PCHSSR19	Nishitani *et al*. (2009)	(CT)_16_._5_	55	Pear
PCHSSR27	Yamamoto *et al*. (2002b)	(AG)_19_	52	Pear
PCHSSR31	Liebhard *et al*. (2002)	PERF	52	Apple

### Genetic distance and clustering

Genetic distance was estimated by analysing dissimilarity indices calculated using allelic data by simple allele matching to obtain the genetic dissimilarity matrix. Dendrogram trees were obtained using Dissimilarity Analysis and Representation for Windows software version 5.0 (DARwin5) by the neighbour-joining method [41]. The robustness of branches was tested using 1,000 bootstraps.

The numbers of genotypes, the numbers of alleles, the major allele frequency (MAF), the expected heterozygosity (exp-het), the observed heterozygosity (obs-het) and the polymorphism information content (PIC value) for each SSR marker were calculated using PowerMarker [42]. Pairwise fixation index (F_ST_) was calculated using GenePop software [43].

The level of genetic stratification within the germplasms in the analysis was assessed using STRUCTURE v.2.3.1 [44]. This analysis was performed on 73 genotypes, excluding those genotypes for which a third allele was observed for one or more loci. Eight nSSRs were used to compute the posterior probability [Pr(X|K)] given an increasing number of sub-populations (ranging from K = 1 to K = 8, with five independent runs each). The computation was performed with five independent runs using a ‘Length of Burnin Period’ and ‘Number of MCMC Reps after Burnin’ of 1,000,000 under the admixture model. The most likely number of sub-populations (K) was identified with STRUCTURE HARVESTER [45] using the ΔK described by Evanno et al. [46]. Samples were assigned to the sub-population when the assignation probability (*qI*) was greater than or equal to 0.8 [47–49]. Principal component analysis (PCA) was performed using the ‘stat’ package in R (R developing team), whereas median-joining network analyses were performed using Network 4.6.1.5 ([50] http://www.fluxus-engineering.com/sharenet.htm) with default settings.

## Results

Capillary electrophoresis analysis produced clear profiles for all four cpSSR and eight nSSR loci for 91 pear genotypes. In contrast, two local (‘Savino’ and ‘Angelico’) and two wild (*P*. *pyraster* n. 6 and *P*. *amygdaliformis* n. 10) genotypes exhibited no PCR amplification and were excluded from further analysis.

Nuclear SSR markers allowed the identification of nineteen individuals exhibiting three alleles in at least one of the nSSRs (data not shown).

The nSSRs detected a total of 136 alleles with sizes ranging from 115 to 256 bp with average values of 17 and 0.29 for the number of alleles and the MAF, respectively (Table 3). The mean value of the exp-het was 0.82, whereas the obs-het was 0.65. All eight nSSRs were highly polymorphic, with PIC values ranging from 0.42 to 0.92. The most polymorphic markers were TsuENH026 with a total of 21 alleles and 48 genotypes detected, obs-het of 0.74 and PIC of 0.92 and BGT23b with a total of 25 alleles and 43 genotypes, obs-het of 0.55 and PIC of 0.91. The least informative nSSR was CH04e03 demonstrating an obs-het and PIC of 0.18 and 0.42, respectively.

**Table 3:** Polymorphism information for the nuclear and chloroplast markers.

Marker	Size range (bp)	Major allele frequency	Number of genotypes	Number of alleles	Expected heterozygosity	Observed heterozygosity	PIC
NH013a	181–243	0.38	41	18	0.82	0.72	0.81
CH02h11a	129–159	0.20	34	12	0.87	0.71	0.86
BGT23b	193–233	0.16	43	25	0.92	0.55	0.91
CH05d04	190–220	0.27	35	21	0.86	0.76	0.85
TsuENH025	207–256	0.16	39	16	0.90	0.76	0.90
TsuENH026	156–193	0.16	48	21	0.92	0.74	0.92
NH015a	115–153	0.26	32	13	0.84	0.77	0.82
CH04e03	198–226	0.72	12	10	0.45	0.18	0.42
Mean	-	0.29	35.5	17	0.82	0.65	0.81
PCHSSR3*	197–216	0.71	5	5	0.45	-	0.39
PCHSSR19*	199–200	0.69	2	2	0.43	-	0.34
PCHSSR27*	195	1.00	1	1	0.00	-	0.00
PCHSSR31*	182–184	0.92	3	3	0.14	-	0.13
Mean	-	0.83	2.75	2.75	0.25	-	0.22

* Chloroplast markers

The cpSSR analysis detected a total of 11 alleles with an average value of 2.75 alleles and sizes ranging from 182 to 216 bp (Table 3). The chloroplast marker PCHSSR27 was monomorphic, detecting an allele of 195 bp. In contrast, the highest number of alleles (5) was detected for the PCHSSR3 marker. The MAF ranged from 0.69 (PCHSSR19) to 0.92 (PCHSSR31), and PIC values ranged from 0.13 (PCHSSR31) to 0.39 (PCHSSR3).

Overall, cpSSRs and nSSRs discriminated 81 of the 91 analysed genotypes, detecting a total of 147 alleles with an average value of 12.25 and an average MAF of 0.47. The mean values of exp-het, obs-het and PIC were 0.63, 0.43 and 0.61, respectively.

**Fig 2:**
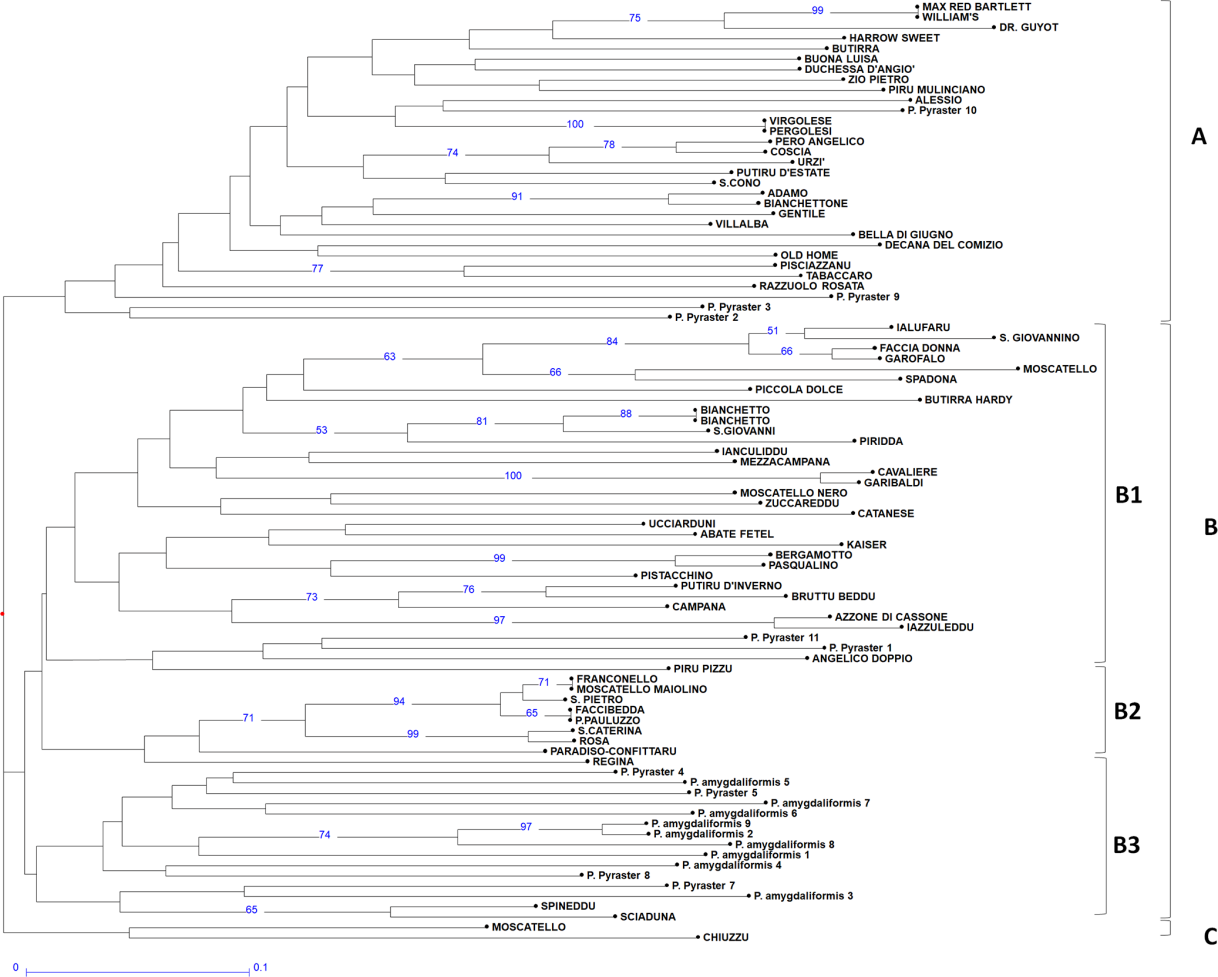
Neighbour-joining analysis. Nj dendrogram calculated by employing both nSSR and cpSSR. The three major clusters (named A, B and C) are reported.

The genetic relationship among analysed genotypes is presented in the neighbour-joining dendrogram constructed using both nSSR and cpSSR data (Fig 2). The cluster analysis identified three main clusters (A-B-C). Cluster A includes six internationally common pear varieties, including ‘Max Red Bartlett’, ‘William’s’, ‘Dr. Guyot’, ‘Harrow Sweet’, ‘Decana del Comizio’ and ‘Old Home’; seven NCV; 14 LV and four RS (*P*. *pyraster* n. 2, 3, 9 and 10). Among these, ‘Max Red Bartlett’, ‘William’s’ and ‘Virgolese’-’Pergolesi’ were undistinguished.

Cluster B includes three subgroups (B1-B2-B3). The first subgroup B1 includes three international varieties, ‘Butirra Hardy’, ‘Abate Fetel’ and ‘Kaiser’; nine NCV; 20 LV; and 2 RS (*P*. *pyraster* n. 1 and 11). Among these, the two ‘Bianchetto’ accessions (1 and 2) presented the same SSR profile. The second subgroup includes nine local genotypes, among which ‘Faccibedda’-’Pauluzzo’ and ‘Moscatello maiolino’-’Franconello’ exhibited the same SSR profile. The third subgroup includes all nine wild genotypes of *P*. *amygdaliformis*, four wild genotypes of *P*. *pyraster* (n. 4, 5, 7 and 8) and the genotypes ‘Spineddu’ (NCV) and ‘Sciaduna’ (LV). The local genotypes ‘Moscatello’ (NCV) and ‘Chiuzzu’ (LV) represented cluster C.

The 73 genotypes exhibiting one or two alleles for each locus were also included in a population stratification analysis. Unlike the results of the neighbour-joining analysis, the population stratification analysis identified two sub-populations (K = 2). This analysis was performed following a plateau criterion [51], a non-parametric Wilcoxon test [52], and the rate of change (ΔK) method proposed by Evanno et al. [46] (S2 Table).

**Fig 3:**
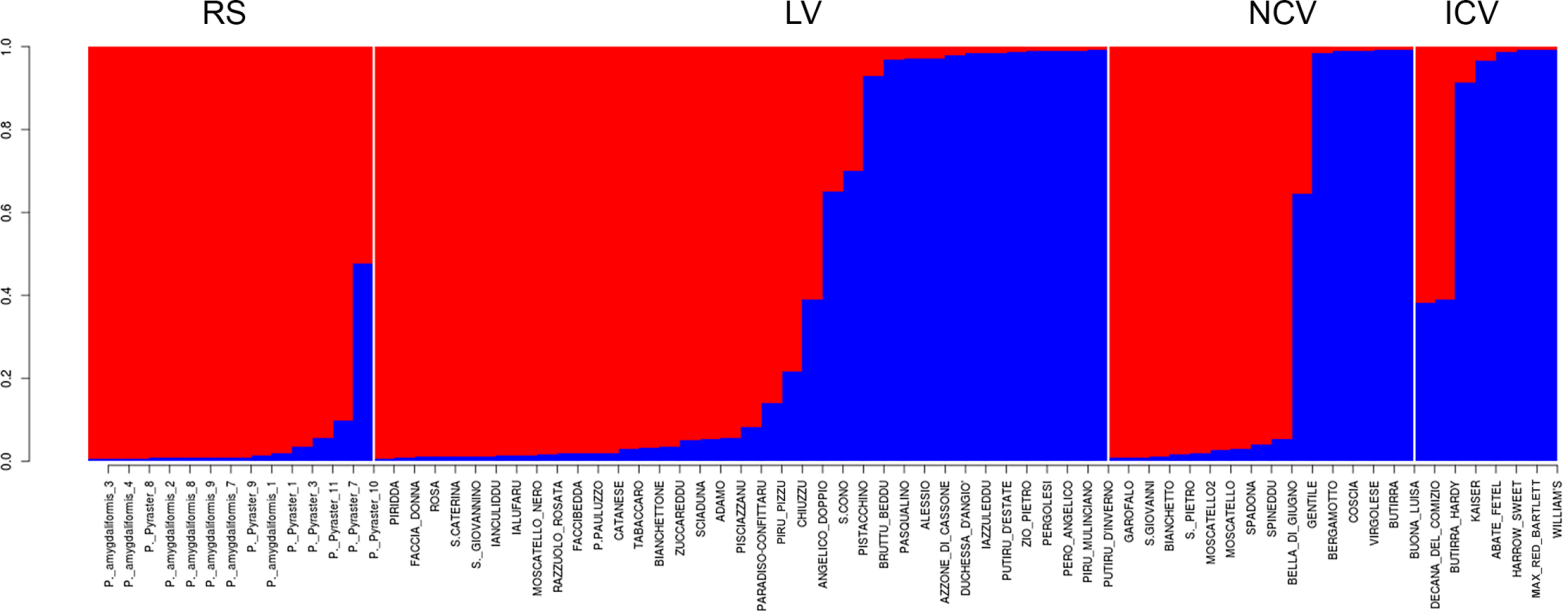
STRUCTURE results. Inferred population structure: Bar plot generated by STRUCTURE according to the K = 2 model based on eight nSSRs.

STRUCTURE analysis allowed the identification of two groups that will be henceforth named ‘wild’ and ‘cultivated’ sub-populations (Fig 3, S3 Table). Forty-two samples were characterized by a predominant (*QI* > 0.8) ‘wild’ genetic configuration, whereas twenty-two exhibited a predominance of the ‘cultivated’ sub-population. The remaining nine samples exhibited *QI* values less than 0.8 for both subpopulations and were therefore considered ‘admixed’ (S3 Table).

Within the four groups of pears (Table 1), individuals exhibited different relative frequencies of the ‘wild’ and ‘cultivated’ sub-populations. In particular, RS and ICV are mostly characterized by ‘wild’ and ‘cultivated’ genetic configurations, respectively, whereas LV and NCV presented a more balanced presence of both sub-populations (S3 Table). The LV group is characterized by a high relative contribution of the ‘wild’ genetic configuration. In total, 54% of the cultivars within this group exhibited a predominance of ‘wild’ subpopulation, whereas a notable proportion of individuals (32%) exhibited a clear predominance of the ‘cultivated’ subgroup. The same pattern registered in the NCV with nine individuals (60%) exhibiting a strong relative contribution of the ‘wild’ subpopulation, five individuals (33%) exhibiting an opposite trend in favour of the ‘cultivated’ subpopulation, and the remaining sample, ‘Gentile’, exhibiting a more balanced admixture between the two sub-populations (S3 Table).

The distinction between ‘wild’ and ‘cultivated’ subpopulations was further confirmed by the analysis of the fixation index (F_ST_), a summary statistic quantifying the variation in allelic frequencies between groups. The F_ST_ between these two subpopulations was 0.096, whereas the pairwise F_ST_ estimates between ‘admixed’ and ‘wild’ or ‘admixed’ and ‘cultivated’ exhibited considerably reduced values (0.028 and 0.026, respectively).

To examine the presence of additional genetic stratification, the germplasm collection was divided into two subsets based on the two sub-populations detected (‘wild’ and ‘cultivated’), and an additional round of structure analysis was separately performed on each of the two subsets following the approach presented by Urrestarazu and colleagues (2012) [49].

This nested structure analysis allowed for better characterization of each sub-population. The ‘wild’ subpopulation (S3 Table) exhibited the highest ΔK for K = 5 (43.1) although a secondary peak was detected for K = 2 (21.2, S1 Fig). For K = 2, all the *P*. *pyraster* and *P*. *amygdaliformis* accessions were assigned to the same subpopulation. In contrast, at K = 5, the two species were assigned to different sub-populations (‘pink’ for *P*. *pyraster* and ‘yellow’ for *P*. *amygdaliformis*, S2 Fig).

**Fig 4:**
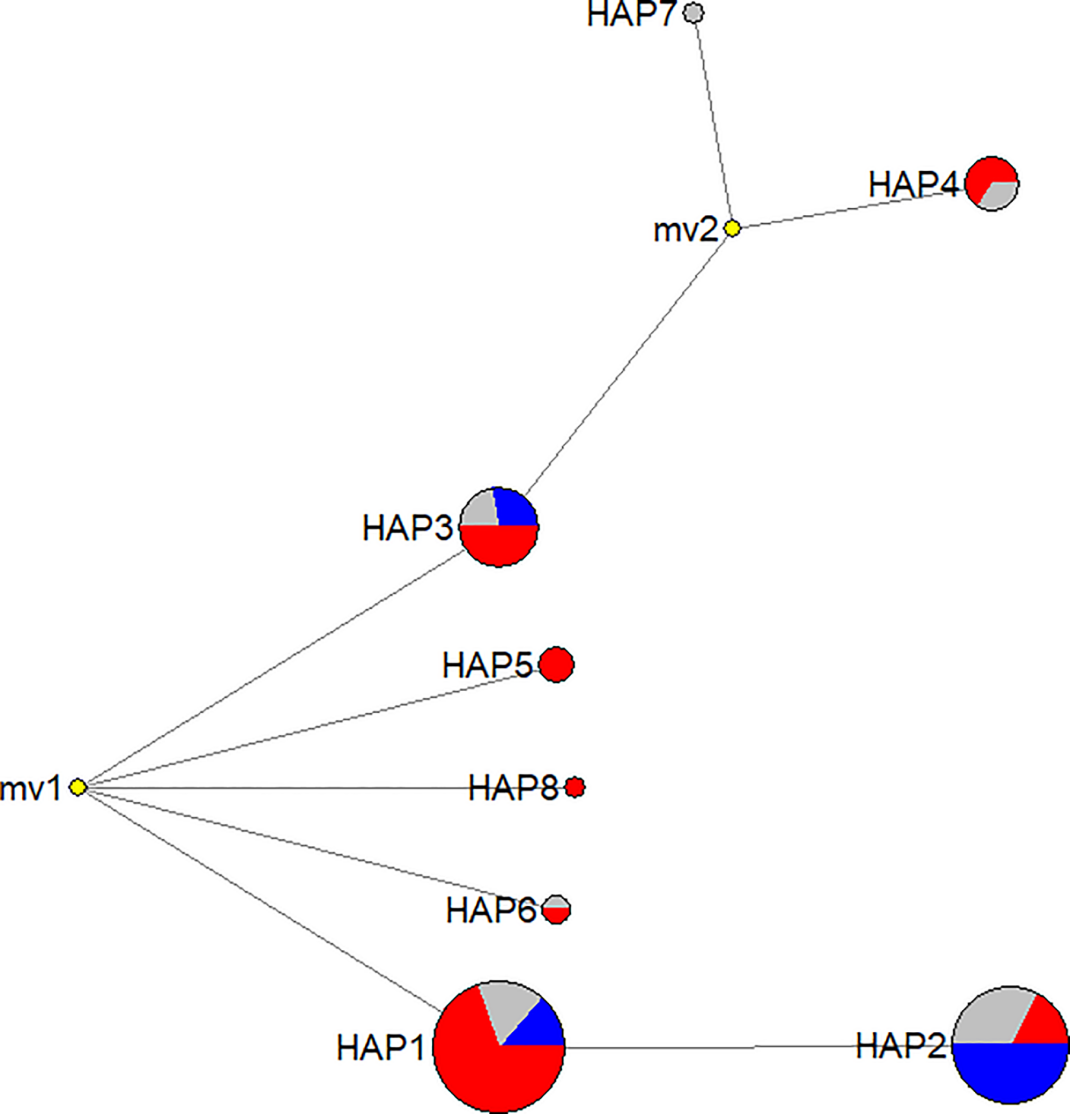
Median-joining network analysis reveals relationships among individuals. Haplotypes have been calculated based on the four cpDNA markers. The pie charts highlight the relative frequency of individuals for each haplotype, exhibiting a clear prevalence (>90%) of one subpopulation (red = wild subpopulation, blue = cultivated subpopulation). Grey represents samples with more evident genetic admixtures.

The 22 samples unambiguously assigned to the ‘cultivated’ subpopulation exhibited similar high ΔK values for K varying from 3 to 4 (S1 Fig). In both scenarios, four samples (‘Bruttu_Beddu’, ‘Putiru_D’Inverno’, ‘Azzone di Cassone’ and ‘Iazzuleddu’) exhibited a clear predominance (*QI* greater than 0.95 and 0.96 for K equal to 3 and 4, respectively, S2 Fig) of one sub-population. The same subpopulation was almost absent in the other samples (*QI* less than 0.07 and 0.08 for K equal to 3 and 4, respectively, S2 Fig). The cpSSRs of the 91 individuals were employed for a median-joining network analysis to further elucidate genetic distances among individuals. The results presented in Fig 4 reveal the presence of nine different haplotypes originating from the combination of the four cpSSRs. The three most common haplotypes (Hap1, Hap2 and Hap3) represent 85.5% of the individuals, accounting for 39.5%, 30.7% and 15.3% of the total genotypes, respectively. The root of the tree (mv1) represents a polymorphism occurring at PCHSSR27, in which one cultivar (‘Kaiser’) exhibiting an allelic size of 194 bp can be considered the origin of the median-joining network, whereas all other individuals exhibit an allelic length of 195 bp (S1 Table). Hap 1, 3, 5, 6 and 8 were further distinguished by different allelic sizes at PCHSSR3, whereas Hap2 originated from Hap1, exhibiting an additional mutation at PCHSSR19. Hap 4 and 7 contain 2 different mutations at PCHSSR31 compared with Hap 3.

**Fig 5:**
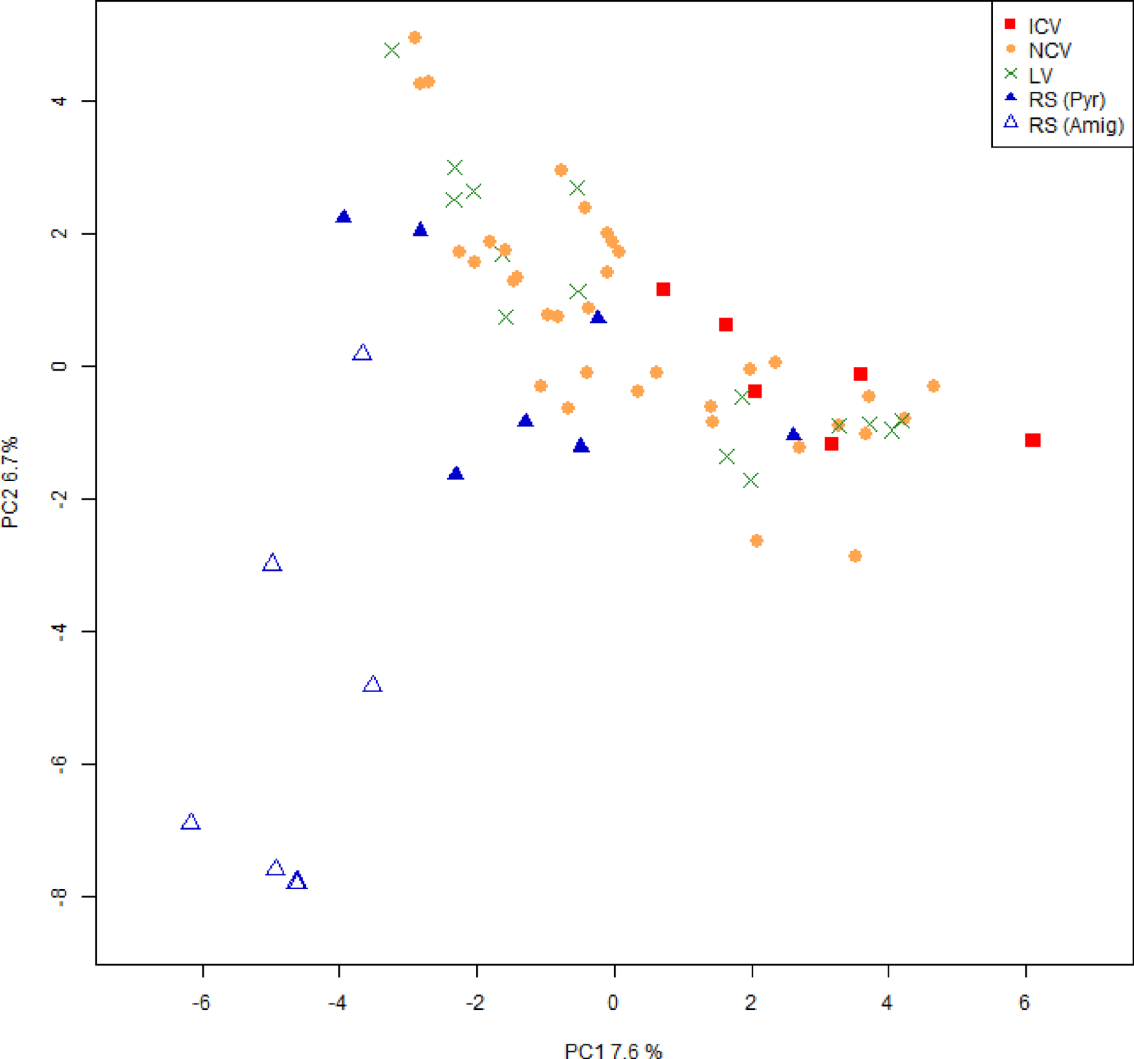
PCA analysis. Two-dimensional PCA plot depicting the distribution of accessions over the first two PCs. Different colours represent the four groups of pears: international cultivar varieties (ICV, red squares), national cultivar varieties (NCV, orange dots), local varieties (LV, green crosses), and wild related species (RS, blue triangles). RS are further subdivided into *P*. *pyraster* (filled triangles) and *P*. *amygdaliformis* (empty triangles).

Both nSSR and cpSSR were employed for PCA. The first two principal components (PC1 and PC2) accounted for 14.5% of total genotypic variability, allowing a distinction of cultivars according to their genetic makeup. The four groups of cultivars defined in Table 1 presented a characteristic pattern on the biplot presented in Fig 5. International cultivars (ICV) were plotted on the positive PC1 quadrant, whereas all wild species (RS) exhibited negative PC1 values with the exception of *P*. *pyraster* 10 (plotted on the positive PC1 quadrant). Local cultivars (LV) and national cultivars (NCV) exhibited a similar pattern characterized by a unimodal distribution of the samples centred in the range between -2 and +2 PC1 values. PC2 allowed a distinction within the RS, NCV and LV groups. The RS group is composed of individuals belonging to *P*. *amygdaliformis* and *P*. *pyraster* species. Extreme negative values indicated *P*. *amygdaliformis* individuals, whereas values of PC2 greater than -3.5 indicated the presence of *P*. *pyraster*. Similarly, NCV and LV can be further differentiated according to PC2, whereas PC3 can efficiently discriminate within the ICV group (data not shown).

## Discussion

The Mount Etna area claims a large number of differentiated pear varieties due to ancient cultivation practises and the variability of soil and climatic conditions. Local varieties display significant variability in many agronomic traits, including fruit size, flowering and ripening periods and harvesting time [53]. Local germplasm could represent an important source of genetic diversity that can be readily used by breeders to develop novel cultivars with enhanced agronomic traits, including fruit quality, adaptability to limiting environmental factors and resistance to biotic stresses. The *Pyrus* genus includes important cultivated species that have been widely studied by means of different molecular markers, mostly nuclear markers. Polyploidy was occasionally observed in several *Pyrus* species [33]. In this work, the phylogenetic relationships among the traditional and broadly cultivated genotypes, wild accessions and related species in *Pyrus* were inferred by coupling cytoplasmic and nuclear markers. Two different types of SSR markers were adopted to combine the investigation of the maternal inheritance property of cpSSRs with the high informativity of nSSRs [54].

The study presented here employed eight nSSRs (Table 2). Among these nSSRs, three (CH02 h11a, CH05d04, CH04e03) were originally developed for apple. The high conservation between the genera *Pyrus* and *Malus* is confirmed by the high heterozygosity of CH02h11a and CH05d04 with the latter exhibiting the highest allelic diversity within the nSSR marker set. On the other hand, CH04e03 exhibited the lowest heterozygosity but was still efficiently used in the germplasm characterization analysis [55]. Although SSRs are not the markers of election for High Throughput Analysis, the use of both nuclear and cytoplasmic microsatellites allowed a high level of discrimination of the collection. Our analysis identified four multi-locus nSSRs (BGT23b, TsuENH025, TsuENH026, NH015a), which is consistent with data previously reported in other studies on genetic characterization in pear [25, 56–58]. The most likely explanation of the high occurrence of multi-locus SSRs can be traced to the allopolyploid origin of Maloideae that originated from ancestors belonging to the subfamily of Spiraeoideae [26, 33, 59–60]. The molecular weight of the amplified fragments, including the M13 tail, falls in the same range of the amplicons obtained in *P*. *communis* for nSSRs [16–17, 55] and *P*. *ussuriensis* for cpSSRs [38]. Overall, the average number of alleles (17) among the analysed genotypes was increased in this study compared with that observed in earlier studies performed in several collections of *P*. *communis* using different sets of SSRs (ranging from 5.9 to 12.8) [21, 25, 54, 56–57, 61–66].

Molecular characterization using SSR markers highlighted high genetic variability among the analysed accessions. It was possible to characterize most of the local varieties and to highlight the genetic relatedness of Italian varieties (NCV + LV) with wild genotypes (Fig 2).

As expected, the international varieties ‘Williams’ and ‘Max Red Bartlett’ exhibited the same SSR profile, confirming the origin of ‘Max Red Bartlett’ as a bud mutation of ‘Williams’. Fingerprint analysis confirmed other previously known pedigree records, such ‘Harrow Sweet’ (‘William’ x ‘Purdue 80–50’), for which the observed SSR profiles were consistent with the known origin. Some local varieties exhibited the same SSR profile (‘Virgolese’-’Pergolesi’; ‘Faccibedda’-’Pauluzzo’; the small-fruit pear ‘Moscatello maiolino’-’Franconello’; the two accessions of ‘Bianchetto’ n. 1 and 2), indicating possible cases of synonymy or, at the very least, a close genetic relationship. This question could be further investigated through phenotypization and/or deeper molecular analysis.

Interestingly, most of the local varieties clustered together in B1 and B2 subclusters of the dendrogram. In structure analysis, most of the local varieties exhibited an increased contribution of the ‘wild’ subpopulation to their genetic makeup.

Among the wild species, few *P*. *pyraster* genotypes were reported in every cluster, exhibiting increased genetic similarity to the local pear varieties compared with *P*. *amygdaliformis*. In contrast, *P*. *amygdaliformis* appeared to be more conserved.

The use of nSSRs in structure analysis (Fig 3) allowed the definition of two sub-populations, defining wild species on one side with all the accessions of *P*. *amygdaliformis* and *P*. *pyraster* and a second group of cultivated pears. The relative contribution of one of the two sub-populations clearly demonstrates the different genetic structure of the ICV and RS groups characterized by a predominant contribution of the ‘cultivated’ and ‘wild’ sub-populations, respectively. In contrast, increased levels of admixture are registered for NCV and LV groups. These results suggest an increased contribution of the ‘wild’ subpopulation to the genetic makeup of many Italian varieties, especially local varieties, compared with internationally cultivated varieties. The F_ST_ between the two subpopulations was 0.096, revealing increased population differentiation compared with other studies on pear germplasm collected in Spain [67] or Bosnia and Herzegovina [68].

The low polymorphism observed within the cpSSRs reflects an increased level of conservation of chloroplast DNA, which is consistent with that previously reported in the wild *P*. *ussuriensis* population [38]. Although none of the represented cpSSRs haplotypes can be unequivocally traced to wild or cultivated pear accessions, Hap2 seems to be more associated with cultivated accessions (89% of samples are cultivated accessions or admixed). In contrast, Hap1 and Hap3 are more associated with wild species (83% and 71%, respectively). Hap4 is exclusively associated with wild or admixture genotypes. However, limited individuals (6) are represented by this haplotype, thus preventing firm conclusions from being drawn (Fig 4). The absence of a haplotype unequivocally associated with wild or cultivated pear is congruent with the high level of admixture within the genus *Pyrus*. Additionally, the lack of a haplotype unequivocally representing one of the two sub-populations (Fig 3) clearly testifies to the occurrence of allelic interchange between wild and cultivated accessions.

Within related species, a different level of genetic admixture was observed. For example, *P*. *amygdaliformis* accessions were characterized by the absence of ‘cultivated’ subpopulation contribution compared with *P*. *pyraster* accessions. In particular, the close genetic proximity of *P*. *pyraster* to most of the local varieties could be explained by its wide employment as rootstock given its rusticity. These findings were further confirmed by our PCA analysis (Fig 5), in which *P*. *pyraster* accessions were located in the upper portion of the plot in the same region as several LV and ICV. In contrast, *P*. *amygdaliformis* accessions were mostly present in the lower left portion of the plot. PCA results were consistent with the outcome of the nested-structure approach. In the first round of structure analysis, both *P*. *pyraster* and *P*. *amygdaliformis* exhibited a predominant contribution of the ‘wild’ subpopulation, which is consistent with that observed in the PCA analysis in which these accessions were characterized by negative PC1 values. *P*. *pyraster* and *P*. *amygdaliformis* can be fully characterized through a second round of structure analysis (at K = 5) or considering the second PC.

## Conclusions

For pear, the identification of traits of agronomic importance, including adaptability to different pedoclimatic conditions and resistance to biotic stresses, is crucial for breeding new varieties. Most of these traits can be found in local germplasm that to date has been less exploited for genetic improvement programmes. The Mount Etna area represents an important biodiversity repository for many woody species due to the presence of many climatic and pedological conditions and a wide range of altitudinal levels. In the case of cultivated species, such as pear, this richness has also been increased by the long history of diffusion and cultivation. In the present work, a set of ninety-five individual trees representing local varieties, wild genotypes and nationally and internationally cultivated varieties have been genotyped to obtain novel insights into pear genetic structure and to elucidate the influence of wild species on the genetic makeup of local germplasm. The molecular insight presented in this work could also be useful for future association studies at the genomic level, such as Genome Wide Association Studies (GWAS), in which a prior knowledge of the genetic diversity of the germplasm collection is a prerequisite of paramount importance.

Overall, our results allow a better understanding of the variability of the cultivars from the Etna area. We have provided evidence of allelic interchange between wild and cultivated sub-populations and provided useful information for better management and conservation of germplasm and the direction of pear breeding programmes.

## Supporting information

S1 Fig Delta_k Log likelihood curve (ΔK) plotted against increasing K value. A: Plot of the complete pear collection, B: Plot for the substructure analysis of the ‘wild’ accessions, C: Plot for the substructure analysis of the ‘cultivated’ accessions.
(TIF)

S2 Fig **Nested structure analysis for the ‘wild’ (A, B) and ‘cultivated’ (C, D) groups.** For each analysis, the results according to the two most likely K value are reported: ‘wild’ K = 2 (A), ‘wild’ K = 5 (B), ‘cultivated’ K = 3 (C), ‘cultivated’ K = 4 (D).
(TIF)

S1 Table Localization of the analysed genotypes used in this study. (DOCX)

S2 Table Descriptive statistics of structure analysis. Structure descriptive statistics. For increasing values of K, the number of replications are reported together with the mean and standard deviation of the estimated Ln probability of data, the mean rate of change of the likelihood distribution Ln'(K), the mean absolute value of the 2^nd^ order rate of change of the likelihood distribution |Ln''(K)| and the ΔK (mean (|Ln''(K)|) / sd(L(K)).
(XLSX)

S3 Table Assigned *QI* values at K = 2. Individuals exhibiting both *QI* values less than 0.8 were considered ‘admixed’. All other individuals are grouped as ‘wild’ or ‘cultivated’ according to the predominant subpopulation. The status of the individual is also reported (RSA = Relative Species Amygdaliformis, RS = Relative Species, LV = Local Varieties, NCV = National Cultivated Varieties, ICV = International Cultivated Varieties).
(XLSX)

S4 Table Haplotypes definition. The four cpDNA markers allowed the definition of eight haplotypes named with progressive numbers from 1 to 8. Genotypes of the single cpDNA markers are reported in the last four columns.
(DOCX)

## References

[1] Continella G, Catalano M, Continella A, La Rosa G, Cicala A, Las Casas G (2006) Recupero di germoplasma di pomacee nel comprensorio etneo. Italus Hortus 13(2): 210–214.

[2] Rosyara UR, Bink MCAM, van de Weg E, Zhang G, Wang D, Sebolt A, et al (2013) Fruit size QTL identification and the prediction of parental QTL genotypes and breeding values in multiple pedigreed populations of sweet cherry. Mol Breeding 32: 875–887.

[3] Paganová V (2003) Wild pear *Pyrus pyraster* L. Burgsd. requirements on environmental conditions. Hortic Sci 22: 225–241.

[4] Allard A, Bink MCAM, Martinez S, Kelner JJ, Legave JM, di Guardo M, et al (2016) Detecting QTLs and putative candidate genes involved in budbreak and flowering time in an apple multiparental population. Journal of Experimental Botany 67: 2875–2888. doi: 10.1093/jxb/erw130 27034326 10.1093/jxb/erw130 PMC4861029

[5] Fresnedo-Ramírez J, Frett TJ, Sandefur PJ, Salgado-Rojas A, Clark JR, Gasic K, et al (2016) QTL mapping and breeding value estimation through pedigree-based analysis of fruit size and weight in four diverse peach breeding programs. Tree Genetics & Genomes 12: 25.

[6] Iketani H, Manabe T, Matsuta N, Akihama T, Hayashi T (1998) Incongruence between RFLPs of chloroplast DNA and morphological classification in east Asian pear (*Pyrus* spp.). Genet Resour Crop Evol 45: 533–539.

[7] Katayama H, Uematsu C (2003) Comparative analysis of chloroplast DNA in *Pyrus* species: physical map and gene localization. Theor Appl Genet 106: 303–310. doi: 10.1007/s00122-002-1003-4 12582856 10.1007/s00122-002-1003-4

[8] Teng Y, Tanabe K, Tamura F, Itai A (2001) Genetic relationships of pear cultivars in Xinjiang, China, as measured by RAPD markers. J Hort Sci Biotechnol 76: 771–779.

[9] Teng Y, Tanabe K, Tamura F, Itai A (2002) Genetic relationships of Pyrus species and cultivars native to East Asia revealed by randomly amplified polymorphic DNA markers. J Am Soc Hortic Sci 127: 262–270.

[10] Monte-Corvo L, Cabrita L, Oliveira C, Leitao J (2000) Assessment of genetic relationships among *Pyrus* species and cultivars using AFLP and RAPD markers. Genet Resour Crop Evol 47: 257–265.

[11] Oliveira CM, Mota M, Monte-Corvo L, Goulao L, Silva DM (1999) Molecular typing of *Pyrus* based on RAPD markers. Sci Hortic 79: 163–174.

[12] Koushesh Saba M, Arzani K, Rasouli M (2017) Genetic Relationship of Iranian Pear Genotypes with European and Asian Pears as Revealed by Random Amplified Polymorphic DNA Markers. Int Journal of Fruit Sci 17(1): 82–92.

[13] Zarei A, Erfani-Moghadam J, Mozaffari M (2017) Phylogenetic analysis among some pome fruit trees of *Rosaceae* family using RAPD markers. Biotechnology and Biotechnological Equipment 31: 289–298.

[14] Bao L, Kunsong C, Dong Z, Xiugen L, Yuanwen T (2008) An assessment of genetic variability and relationships within Asian pears based on AFLP (amplified fragment length polymorphism) markers. Sci Hortic 116: 374–380.

[15] Monte-Corvo L, Goulao L, Oliveira C (2001) ISSR analysis of cultivars of pear and suitability of molecular markers for clone discrimination. J Am Soc Hortic Sci 126: 517–522.

[16] Yamamoto T, Kimura T, Sawamura Y, Manabe T, Kotobuki K, Hayashi T, Ban Y, Matsuta N (2002a) Simple sequence repeats for genetic analysis in pear. Euphytica 124: 129–137.

[17] Yamamoto T, Kimura T, Shoda M, Ban Y, Hayashi T, Matsuta N (2002b) Development of microsatellite markers in the Japanese pear (Pyrus pyrifolia Nakai). Molecular Ecology Notes 2: 14–16.18.

[18] Nishitani C, Terakami S, Sawamura Y, Takada N, Yamamoto T (2009) Development of novel EST-SSR markers derived from Japanese pear (*Pyrus pyrifolia*). Breed Sci 59: 391–400.

[19] Kimura T, Shi YZ, Shoda M, Kotobuki K, Matsuta N, Hayashi T, Ban Y, Tamamoto T (2002) Identification of Asian pear varieties by SSR analysis. Breed Sci 52: 115–121.

[20] Bao L, Chen K, Zhang D, Cao Y, Yamamoto T, Teng Y (2007) Genetic diversity and similarity of pear (Pyrus L.) cultivars native to East Asia revealed by SSR (simple sequence repeat) markers. Genet Resour Crop Evol 54: 959–971.

[21] Bassil NV, Postman J (2009) Identification of European and Asian pears using EST-SSRs from *Pyrus*. Genet Res Crop Evol 57: 357–370.

[22] Rana JC, Chahota RK., Sharma V, Rana M, Verma N, Verma B, et al (2015) Genetic diversity and structure of *Pyrus* accessions of Indian Himalayan region based on morphological and SSR markers. Tree Genet and Genomes 11: 821.

[23] Yamamoto T, Kimura T, Sawamura Y, Kotobuki K, Ban Y, Hayashi T, Matsuta N (2001) SSRs isolated from apple can identify polymorphism and genetic diversity in pear. Theor Appl Genet 102: 865–870.

[24] Liebhard R, Gianfranceschi L, Koller B, Ryder CD, Tarchini R, et al (2002) Development and characterization of 140 new microsatellites in apple (*Malus x domestica* Borkh.). Mol Breed 10: 217–24.

[25] Wünsch A, Hormaza JI (2007) Characterization of variability and genetic similarity of European pear using microsatellite loci developed in apple. Sci Hortic 113: 37–43.

[26] Campbell CS, Evans RC, Morgan DR, Dickinson TA, Arsenault MP (2007) Phylogeny of subtribe Pyrinae (formerly the Maloideae, Rosaceae): limited resolution of a complex evolutionary history. Pl Syst Evol 266: 119–145.

[27] Katayama H, Tachibana M, Iketani H, Zhang SL, Uematsu C (2012) Phylogenetic utility of structural alterations found in the chloroplast genome of pear: hypervariable regions in highly conserved genome. Tree Genet & Genomes 8: 313–326.

[28] Liu J, Sun P, Zheng X, Potter D, Li K, Hu C et al (2013) Genetic structure and phylogeography of Pyrus pashia L. (Rosaceae) in Yunnan Province, China, revealed by chloroplast DNA analysies. Tree Genet & Genomes 9: 433–441.

[29] Wuyun T, Ma T, Uematsu C, Katayama H (2013) A phylogenetic network of wild Ussurian pears (Pyrus ussuriensis Maxim.) in China revealed by hypervariable regions of chloroplast DNA. Tree Genet & Genomes 9: 167–177.

[30] Korotkova N, Nauheimer L, Ter-Voskanyan H, Allgaier M, Borsch T (2014) Variability among the most rapidly evolving plastid genomic regions is lineage-specific: implications of pairwise genome comparisons in Pyrus (Rosaceae) and other Angiosperms for marker choice. PLoS One 9(11): e112998 doi: 10.1371/journal.pone.0112998 25405773 10.1371/journal.pone.0112998 PMC4236126

[31] Weising K, Gardner RC (1999) A set of conserved PCR primers for the analysis of simple sequence repeat polymorphisms in chloroplast genomes of dicotyledonous angiosperms. Genome 42: 9–19. 10207998

[32] Xu D, Abe J, Gai J, Shimamoto Y (2002) Diversity of chloroplast DNA SSRs in wild and cultivated soybeans: evidence for multiple origins of cultivated soybean. Theor Appl Genet 105: 645–653. doi: 10.1007/s00122-002-0972-7 12582476 10.1007/s00122-002-0972-7

[33] Yamamoto T, Kimura T, Soejima J, Sanada T, Ban Y, Hayashi T (2004) Identification of quince varieties using SSR markers developed from pear and apple. Breed Sci 54: 239–244.

[34] Provan J, Powell W, Hollingsworth PM (2001) Chloroplast microsatellites: new tools for studies in plant ecology and evolution. Trends Ecol Evol 16: 142–147. 11179578 10.1016/s0169-5347(00)02097-8

[35] Katayama H, Adachi S, Yamamoto T, Uematsu C (2007) A wide range of genetic diversity in pear (Pyrus ussuriensis var. aromatica) genetic resources from Iwate, Japan revealed by SSR and chloroplast DNA markers. Genet Resour Crop Evol 54: 1573–1585.

[36] Zheng X, Cai D, Potter D, Postman J, Liu J, Teng Y (2014) Phylogeny and evolutionary histories of Pyrus L. revealed by phylogenetic trees and networks based on data from multiple DNA sequences. Mol Phylogenet Evol 80: 54–65. doi: 10.1016/j.ympev.2014.07.009 25083939 10.1016/j.ympev.2014.07.009

[37] Terakami S, Matsumura Y, Kurita K, Kanamori H, Katayose Y, Yamamoto T, et al (2012) Complete sequence of the chloroplast genome from pear (Pyrus pyrifolia): genome structure and comparative analysis. Tree Genet & Genomes 8: 1–14.

[38] Wuyun T, Amo H, Xu J, Ma T, Uematsu C, Katayama H (2015) Population Structure of and Conservation Strategies for Wild Pyrus ussuriensis Maxim. in China. PlosOne 10(8): e0133686. 10.1371/journal.pone.0133686 PMC4529180 26252516

[39] Ferradini N, Lancioni H, Torricelli R, Russi L, Ragione ID, et al (2017) Characterization and phylogenetic analysis of ancient Italian landraces of pear. Front Plant Sci 8:751 doi: 10.3389/fpls.2017.00751 28539931 10.3389/fpls.2017.00751 PMC5423897

[40] Reim S, Lochschmidt F, Proft A, Wolf H, Wolf H (2017) Species delimitation, genetic diversity and structure of the European indigenous wild pear (Pyrus pyraster) in Saxony, Germany. Genet Resour Crop Evol 64: 1075–1085.

[41] Saitou N, Nei M (1987) The Neighbor-Joining Method–a new method for reconstructing phylogenetic trees. Mol Biol Evol 4: 406–425. doi: 10.1093/oxfordjournals.molbev.a040454 3447015 10.1093/oxfordjournals.molbev.a040454

[42] Liu K, Muse SV (2005) PowerMarker: an integrated analysis environment for genetic marker analysis. Bioinformatics 21: 2128–2129. doi: 10.1093/bioinformatics/bti282 15705655 10.1093/bioinformatics/bti282

[43] Raymond M, Rousset F (1995) GENEPOP (version 1.2): population genetics software for exact tests and ecumenicism. J Heredity 86: 248–249.

[44] Pritchard JK, Stephens M, Donnelly P (2000) Inference of population structure using multilocus genotype data. Genetics 155: 945–959. 10835412 10.1093/genetics/155.2.945 PMC1461096

[45] Earl DA, vonHoldt BM (2012) Structure Harvester: a website and program for visualizing Structure output and implementing the Evanno method. Conservation Genetics Resources 4: 359–361.

[46] Evanno G, Regnaut S, Goudet J (2005) Detecting the number of clusters of individuals using the software STRUCTURE: a simulation study. Mol Ecol 14: 2611–2620. doi: 10.1111/j.1365-294X.2005.02553.x 15969739 10.1111/j.1365-294X.2005.02553.x

[47] Miranda C, Urrestarazu J, Santesteban LG, Royo JB, Urbina V (2010) Genetic diversity and structure in a collection of ancient Spanish pear cultivars assessed by microsatellite markers. J AmSoc Hortic Sci 135:428–437

[48] Pereira-Lorenzo S, Costa RML, Ramos-Cabrer AM, Ribeiro CAM, da Silva MFS, Manzano G, Barreneche T (2010) Variation in grafted European chestnut and hybrids by microsatellites reveals two main origins in the Iberian Peninsula. Tree Genet Genomes 6: 701–715.

[49] Urrestarazu J, Miranda C, Santesteban LG, Royo JB (2012) Genetic diversity and structure of local apple cultivars from northeastern Spain assessed by microsatellite markers. Tree Genet Genomes 8: 1163–1180.

[50] Bandelt HJ, Forster P, Röhl A (1999) Median-joining networks for inferring intraspecific phylogenies. Mol Biol Evol 16: 37–48. doi: 10.1093/oxfordjournals.molbev.a026036 10331250 10.1093/oxfordjournals.molbev.a026036

[51] Falush D, Stephens M, Pritchard JK (2007) Inference of population structure using multilocus genotype data: dominant markers and null alleles. Mol Ecol Notes 7: 574–578. doi: 10.1111/j.1471-8286.2007.01758.x 18784791 10.1111/j.1471-8286.2007.01758.x PMC1974779

[52] Rosenberg NA, Pritchard JK, Weber JL, Cann HM, Kidd KK, Zhivotovsky LA, et al (2002) Genetic structure of human populations. Science 298: 2981–2985. 10.1126/science.1078311 12493913

[53] Damigella P, Alberghina O (1991) Le cultivar di pero (Pyrus communis L.) di antica diffusione nella Sicilia centro-orientale 39 Quaderni di ricerca e sperimentazione. Publisicula Editrice.

[54] Pleines T, Jakob SS, Blattner FR (2009) Application of non-coding DNA regions in intraspecific analyses. Plant Syst Evol 282: 281–294.

[55] Erfani J, Ebadi A, Abdollahi H, Fatahi R (2012) Genetic Diversity of Some Pear Cultivars and Genotypes Using Simple Sequence Repeat (SSR) Markers. Plant Mol Biol Rep 30: 1065–1072.

[56] Brini W, Mars M, Hormaza JI (2008) Genetic diversity in local Tunisian pears (Pyrus communis L.) studied with SSR markers. Sci Hortic 115: 337–341.

[57] Urbanovich OY, Kazlouvskaya ZA, Yakimovich OA, Kartel NA (2011) Polymorphism of SSR alleles in pear cultivars grown in Belarus. Russ J Genet 47: 305–313. 21542305

[58] Sehic J, Garkava-Gustavsson L, Fernández-Fernández F, Nybom H (2012) Genetic diversity in a collection of European pear (Pyrus communis) cultivars determined with SSR markers chosen by ECPGR. Sci Hortic 145: 39–45.

[59] Evans RC, Campbell CS (2002) The origin of the apple subfamily (Maloideae; Rosaceae) is clarified by DNA sequence data from duplicated GBSSI genes. Am J Bot. 89: 1478–1484. doi: 10.3732/ajb.89.9.1478 21665749 10.3732/ajb.89.9.1478

[60] Velasco R, Zharkikh A, Affourtit J, Dhingra A, Cestaro A, Kalyanaraman A, et al (2010) The genome of the domesticated apple (Malus domestica Borkh.). Nat Genet 42: 833–839. doi: 10.1038/ng.654 20802477 10.1038/ng.654

[61] Fernández-Fernández F, Harvey NG, James CM (2006) Isolation and characterization of polymorphic microsatellite markers from European pear (Pyrus communis L.). Mol Ecol Notes 6: 1039–1041.

[62] Xuan H (2008) Identifying European pear (Pyrus communis L.) cultivars at the KOB by using apple SSRs. Acta Hortic 800: 439–445.

[63] Sisko M, Javornik B, Siftar A, Ivancic A (2009) Genetic relationships among Slovenian pears assessed by molecular markers. J Am Soc Hortic Sci 134: 97–108.

[64] Ahmed M, Anjum MA, Khan MQ, Ahmed MJ, Pearce S (2010) Evaluation of genetic diversity in Pyrus germplasm native to Azad Jammu and Kashmir (Northern Pakistan) revealed by microsatellite markers. Afr J Biotechnol 9: 8323–8333.

[65] Miranda C, Urrestarazu J, Santesteban LG, Royo JB, Urbina V (2010) Genetic diversity and structure in a collection of ancient Spanish pear cultivars assessed by microsatellite markers. J Am Soc Hortic Sci 135: 428–437.

[66] Yakovin NA, Fesenko IA, Isachkin AV, Karlov GI (2011) Polymorphism of microsatellite loci in cultivars and species of pear (Pyrus L.). Russ J Genet 47: 564–570. 21786670

[67] Urrestarazu J, Royo JB, Santesteban LG, Miranda C (2015) Evaluating the Influence of the Microsatellite Marker Set on the Genetic Structure Inferred in Pyrus communis L. Plos ONE 0138417. 10.1371/journal.pone.0138417 PMC4575082 26382618

[68] Gasi F, Kurtovic M, Kalamujic B, Pojskic N, Grahic J, Kaiser C, et al (2013) Assessment of European pear (*Pyrus communis* L.) genetic resources in Bosnia and Herzegovina using microsatellite markers. Sci Hortic 157: 74–83.

