# Supplementary figures and images for: Elucidating the contribution of wild related species on autochthonous pear germplasm: A case study from Mount Etna
Source: PLoS One. 2018 Jun 1;13(6):e0198512. doi: 10.1371/journal.pone.0198512 (PMC5983503; doi:10.1371/journal.pone.0198512)

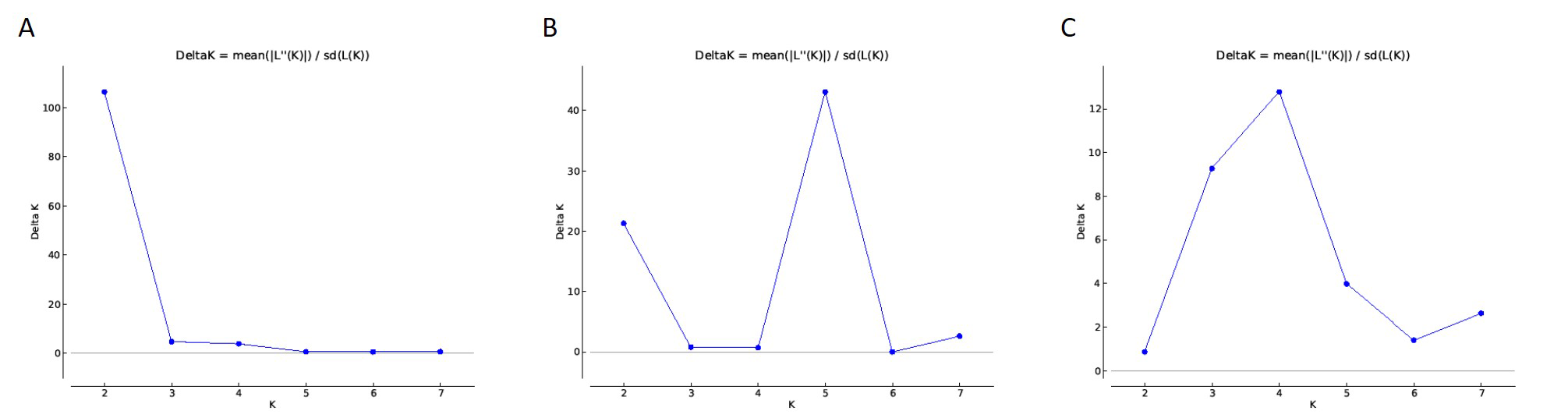

Supplement: S1 Fig — A: Plot of the complete pear collection, B: Plot for the substructure analysis of the ‘wild’ accessions, C: Plot for the substructure analysis of the ‘cultivated’ accessions. (TIF) [file pone.0198512.s001.tif]

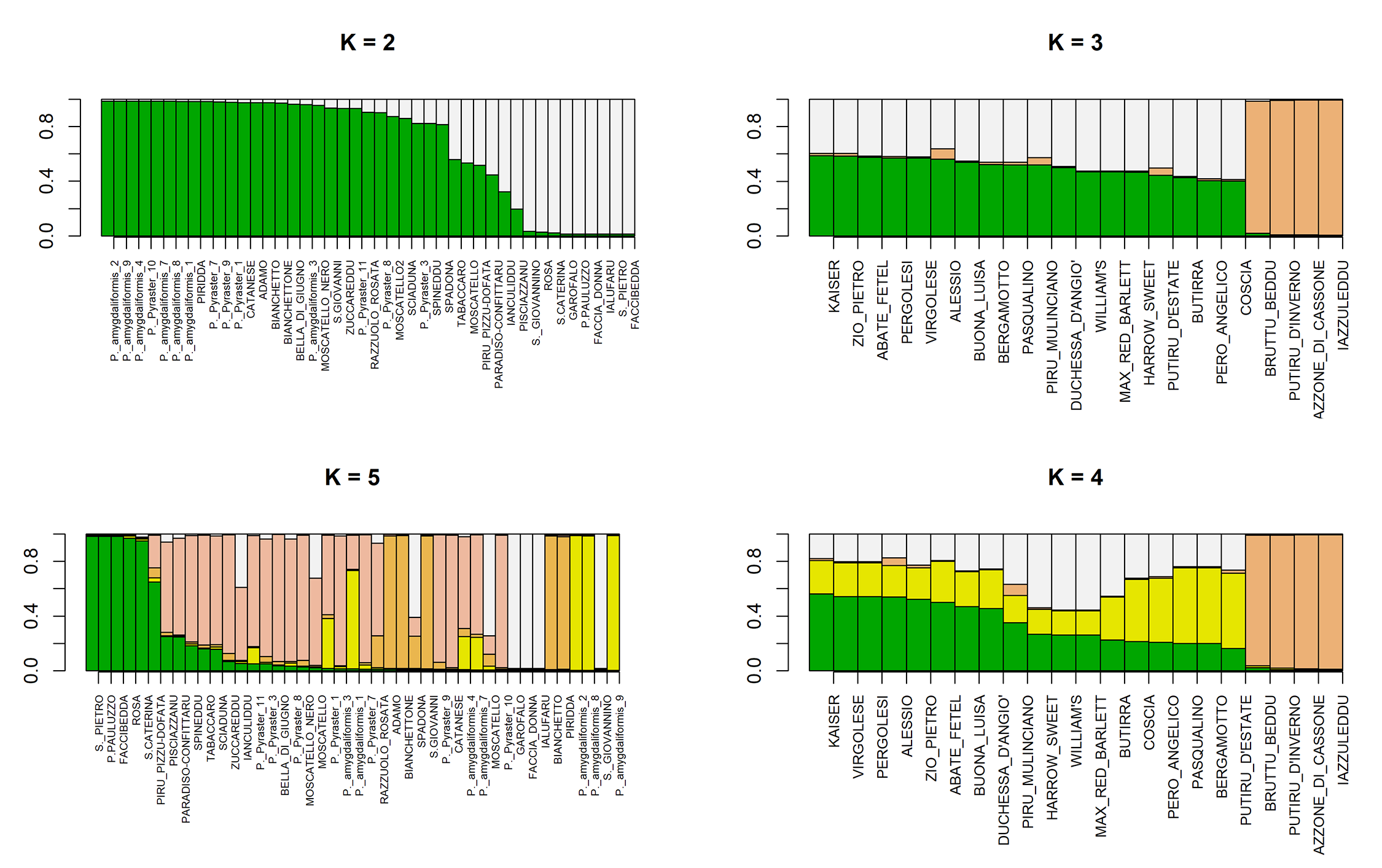

Supplement: S2 Fig — Nested structure analysis for the ‘wild’ (A, B) and ‘cultivated’ (C, D) groups. For each analysis, the results according to the two most likely K value are reported: ‘wild’ K = 2 (A), ‘wild’ K = 5 (B), ‘cultivated’ K = 3 (C), ‘cultivated’ K = 4 (D). (TIF) [file pone.0198512.s002.tif]
