# Supplementary material for: Elucidating the contribution of wild related species on autochthonous pear germplasm: A case study from Mount Etna
Source: PLoS One. 2018 Jun 1;13(6):e0198512. doi: 10.1371/journal.pone.0198512 (PMC5983503; doi:10.1371/journal.pone.0198512)
Supplement: S1 Table — (DOCX) [file pone.0198512.s003.docx]

**Table S1. Localization of the analysed genotypes used in this study.**

| **Species** | **Accession name** | **Status*** | **Origin** | **Collection site**** |
| --- | --- | --- | --- | --- |
| *P. communis* | Adamo | LV | Italy (Sicily) | UNICT |
| *P. communis* | Alessio | LV | Italy (Sicily) | Mt Etna |
| *P. communis* | Angelico | NCV | Italy (Sicily) | Mt Etna |
| *P. communis* | Angelico Doppio | LV | Italy (Sicily) | Mt Etna |
| *P. communis* | Azzone di Cassone | LV | Italy (Sicily) | UNICT |
| *P. communis* | Bella di Giugno | NCV | Italy (Sicily) | Mt Etna |
| *P. communis* | Bergamotto | NCV | Italy (Sicily) | UNICT |
| *P. communis* | Bianchetto (1) | NCV | Italy (Sicily) | UNICT |
| *P. communis* | Bianchetto (2) | NCV | Italy (Sicily) | Mt Etna |
| *P. communis* | Bianchettone | LV | Italy (Sicily) | Mt Etna |
| *P. communis* | Bruttu Beddu | LV | Italy (Sicily) | Mt Etna |
| *P. communis* | Buona Luisa | NCV | Italy (Sicily) | Mt Etna |
| *P. communis* | Butirra | NCV | Italy (Sicily) | UNICT |
| *P. communis* | Campana | NCV | Italy (Sicily) | UNICT |
| *P. communis* | Catanese | LV | Italy (Sicily) | Mt Etna |
| *P. communis* | Cavaliere | LV | Italy (Sicily) | UNICT |
| *P. communis* | Chiuzzu | LV | Italy (Sicily) | UNICT |
| *P. communis* | Coscia | NCV | Italy (Sicily) | UNICT |
| *P. communis* | Duchessa D'angio' | LV | Italy (Sicily) | Mt Etna |
| *P. communis* | Faccia Donna | LV | Italy (Sicily) | UNICT |
| *P. communis* | Faccibedda | LV | Italy (Sicily) | UNICT |
| *P. communis* | Franconello | LV | Italy (Sicily) | Mt Etna |
| *P. communis* | Garibaldi | LV | Italy (Sicily) | Mt Etna |
| *P. communis* | Garofalo | NCV | Italy (Sicily) | Mt Etna |
| *P. communis* | Gentile | NCV | Italy (Sicily) | UNICT |
| *P. communis* | Ialufaru | LV | Italy (Sicily) | Mt Etna |
| *P. communis* | Ianculiddu | LV | Italy (Sicily) | Mt Etna |
| *P. communis* | Iazzuleddu | LV | Italy (Sicily) | UNICT |
| *P. communis* | Mezza Campana | LV | Italy (Sicily) | Mt Etna |
| *P. communis* | Moscatello (2) | NCV | Italy (Sicily) | Mt Etna |
| *P. communis* | Moscatello (1) | NCV | Italy (Sicily) | UNICT |
| *P. communis* | Moscatello Maiolino | LV | Italy (Sicily) | Mt Etna |
| *P. communis* | Moscatello Nero | LV | Italy (Sicily) | Mt Etna |
| *P. communis* | Paradiso/Confittaru | LV | Italy (Sicily) | Mt Etna |
| *P. communis* | Pasqualino | LV | Italy (Sicily) | UNICT |
| *P. communis* | Pauluzzo | LV | Italy (Sicily) | Mt Etna |
| *P. communis* | Pergolesi | LV | Italy (Sicily) | Mt Etna |
| *P. communis* | Pero Angelico | LV | Italy (Sicily) | Mt Etna |
| *P. communis* | Piccola Dolce | LV | Italy (Sicily) | Mt Etna |
| *P. communis* | Piridda | LV | Italy (Sicily) | Mt Etna |
| *P. communis* | Piru Mulinciana | LV | Italy (Sicily) | Mt Etna |
| *P. communis* | Piru Pizzu | LV | Italy (Sicily) | Mt Etna |
| *P. communis* | Pisciazzaru | LV | Italy (Sicily) | Mt Etna |
| *P. communis* | Pistacchino | LV | Italy (Sicily) | Mt Etna |
| *P. communis* | Putiru d'Estate | LV | Italy (Sicily) | Mt Etna |
| *P. communis* | Putiru d'Inverno | LV | Italy (Sicily) | UNICT |
| *P. communis* | Razzuolo Rosata | LV | Italy (Sicily) | Mt Etna |
| *P. communis* | Regina | LV | Italy (Sicily) | UNICT |
| *P. communis* | Rosa | LV | Italy (Sicily) | Mt Etna |
| *P. communis* | San Cono | LV | Italy (Sicily) | Mt Etna |
| *P. communis* | San Giovanni | NCV | Italy (Sicily) | UNICT |
| *P. communis* | San Giovannino | LV | Italy (Sicily) | Mt Etna |
| *P. communis* | San Pietro | NCV | Italy (Sicily) | UNICT |
| *P. communis* | Santa Caterina | LV | Italy (Sicily) | UNICT |
| *P. communis* | Savino | LV | Italy (Sicily) | Mt Etna |
| *P. communis* | Sciaduna | LV | Italy (Sicily) | Mt Etna |
| *P. communis* | Spadona | NCV | Italy (Sicily) | Mt Etna |
| *P. communis* | Spineddu | NCV | Italy (Sicily) | UNICT |
| *P. communis* | Tabaccaro | LV | Italy (Sicily) | Mt Etna |
| *P. communis* | Ucciarduni | NCV | Italy (Sicily) | UNICT |
| *P. communis* | Urzi' | LV | Italy (Sicily) | UNICT |
| *P. communis* | Villalba | LV | Italy (Sicily) | Mt Etna |
| *P. communis* | Virgolese | NCV | Italy (Sicily) | UNICT |
| *P. communis* | Zio Pietro | LV | Italy (Sicily) | UNICT |
| *P. communis* | Zuccareddu | LV | Italy (Sicily) | Mt Etna |
| *P. amygdaliformis* | 1 | RS | Italy (Sicily) | N 37° 47' 692'' E 14° 50' 925'' |
| *P. amygdaliformis* | 2 | RS | Italy (Sicily) | N 37° 51' 189'' E 14° 50' 795'' |
| *P. amygdaliformis* | 3 | RS | Italy (Sicily) | N 37° 51' 147'' E 14° 50' 739'' |
| *P. amygdaliformis* | 4 | RS | Italy (Sicily) | N 37° 51' 109'' E 14°50' 809'' |
| *P. amygdaliformis* | 5 | RS | Italy (Sicily) | N 37° 51' 116'' E 14° 50' 806'' |
| *P. amygdaliformis* | 6 | RS | Italy (Sicily) | N 37° 51' 115'' E 14° 50' 788'' |
| *P. amygdaliformis* | 7 | RS | Italy (Sicily) | N 37° 51' 139'' E 14° 50' 765'' |
| *P. amygdaliformis* | 8 | RS | Italy (Sicily) | N 37° 51' 185'' E 14° 50' 743'' |
| *P. amygdaliformis* | 9 | RS | Italy (Sicily) | N 37° 51' 182'' E 14° 50' 731'' |
| *P. amygdaliformis* | 10 | RS | Italy (Sicily) | N 37° 51' 137'' E 14° 50' 748'' |
| *P. pyraster* | 1 | RS | Italy (Sicily) | N 37° 52' 912'' E 14° 52' 581'' |
| *P. pyraster* | 2 | RS | Italy (Sicily) | N 37° 48' 143'' E 14° 51' 203'' |
| *P. pyraster* | 3 | RS | Italy (Sicily) | N 37° 51' 796'' E 14° 52' 409'' |
| *P. pyraster* | 4 | RS | Italy (Sicily) | N 37° 52' 808'' E 14° 52' 571'' |
| *P. pyraster* | 5 | RS | Italy (Sicily) | N 37° 52' 868'' E 14° 52' 565'' |
| *P. pyraster* | 6 | RS | Italy (Sicily) | N 37° 53' 028'' E 14° 52' 578'' |
| *P. pyraster* | 7 | RS | Italy (Sicily) | N 37° 47' 691'' E 14° 50' 901'' |
| *P. pyraster* | 8 | RS | Italy (Sicily) | N 37° 52' 006'' E 14° 52' 969'' |
| *P. pyraster* | 9 | RS | Italy (Sicily) | N 37° 51' 865'' E 14° 52' 672'' |
| *P. pyraster* | 10 | RS | Italy (Sicily) | N 37° 48' 276'' E 14° 51' 269'' |
| *P. pyraster* | 11 | RS | Italy (Sicily) | N 37° 53' 038'' E 14° 52' 578'' |
| *P. communis* | Abate Fetel | ICV | France | UNIBO |
| *P. communis* | Butirra Hardy | ICV | France | UNIBO |
| *P. communis* | Decana del Comizio | ICV | France | UNIBO |
| *P. communis* | Dr. Jules Guyot | ICV | France | UNIBO |
| *P. communis* | Kaiser | ICV | France | UNIBO |
| *P. communis* | Max Red Bartlett | ICV | USA | UNIBO |
| *P. communis* | Old Home | ICV | USA | UNIBO |
| *P. communis* | Harrow Sweet | ICV | Canada | UNIBO |
| *P. communis* | William’s | ICV | England | UNIBO |

*Accessions status classification:

RS: wild Related Species

LV: Local Varieties

NCV: National Commercial Varieties

ICV: International Commercial Varieties

**Collection site:

UNICT: University of Catania, Catania, Italy, experimental field

Mt Etna: Mount Etna Regional Park, Nicolosi, Italy, collection field

UNIBO: University of Bologna, Bologna, Italy, experimental field
