# Supplementary material for: Elucidating the contribution of wild related species on autochthonous pear germplasm: A case study from Mount Etna
Source: PLoS One. 2018 Jun 1;13(6):e0198512. doi: 10.1371/journal.pone.0198512 (PMC5983503; doi:10.1371/journal.pone.0198512)
Supplement: S4 Table — The four cpDNA markers allowed the definition of eight haplotypes named with progressive numbers from 1 to 8. Genotypes of the single cpDNA markers are reported in the last four columns. (DOCX) [file pone.0198512.s006.docx]

**Table S4. Haplotypes definition.** The four cpDNA markers allowed the definition of eight haplotypes named with progressive numbers from 1 to 8. Genotypes of the single cpDNA markers are reported in the last four columns.

| **Haplotypes** | **Genotypes** | **PCHSSR3** | | **PCHSSR19** | | **PCHSSR27** | **PCHSSR31** |
| --- | --- | --- | --- | --- | --- | --- | --- |
| 1 | BRUTTU BEDDU | | 202 | | 199 | 195 | 182 |
| 1 | ABATE FETEL | | 202 | | 199 | 195 | 182 |
| 1 | CAMPANA | | 202 | | 199 | 195 | 182 |
| 1 | CHIUZZU | | 202 | | 199 | 195 | 182 |
| 1 | FACCIBEDDA | | 202 | | 199 | 195 | 182 |
| 1 | FRANCONELLO | | 202 | | 199 | 195 | 182 |
| 1 | KAISER | | 202 | | 199 | 194 | 182 |
| 1 | MOSCATELLO | | 202 | | 199 | 195 | 182 |
| 1 | MOSCATELLO MAIOLINO | | 202 | | 199 | 195 | 182 |
| 1 | *P. amygdaliformis 1* | | 202 | | 199 | 195 | 182 |
| 1 | *P. amygdaliformis 2* | | 202 | | 199 | 195 | 182 |
| 1 | *P. amygdaliformis 3* | | 202 | | 199 | 195 | 182 |
| 1 | *P. amygdaliformis 4* | | 202 | | 199 | 195 | 182 |
| 1 | *P. amygdaliformis 5* | | 202 | | 199 | 195 | 182 |
| 1 | *P. amygdaliformis 6* | | 202 | | 199 | 195 | 182 |
| 1 | *P. amygdaliformis 8* | | 202 | | 199 | 195 | 182 |
| 1 | *P. amygdaliformis 9* | | 202 | | 199 | 195 | 182 |
| 1 | *P. Pyraster 11* | | 202 | | 199 | 195 | 182 |
| 1 | *P. Pyraster 2* | | 202 | | 199 | 195 | 182 |
| 1 | *P. Pyraster 3* | | 202 | | 199 | 195 | 182 |
| 1 | *P. Pyraster 4* | | 202 | | 199 | 195 | 182 |
| 1 | *P. Pyraster 7* | | 202 | | 199 | 195 | 182 |
| 1 | *P. Pyraster 8* | | 202 | | 199 | 195 | 182 |
| 1 | *P. Pyraster 9* | | 202 | | 999 | 195 | 182 |
| 1 | P.PAULUZZO | | 202 | | 199 | 195 | 182 |
| 1 | PARADISO-CONFITTARU | | 202 | | 199 | 195 | 182 |
| 1 | PIRU PIZZU | | 202 | | 199 | 195 | 182 |
| 1 | PISTACCHINO | | 202 | | 199 | 195 | 182 |
| 1 | PUTIRU D'INVERNO | | 202 | | 199 | 195 | 182 |
| 1 | REGINA | | 202 | | 199 | 195 | 182 |
| 1 | ROSA | | 202 | | 199 | 195 | 182 |
| 1 | S. PIETRO | | 202 | | 199 | 195 | 182 |
| 1 | S.CATERINA | | 202 | | 199 | 195 | 182 |
| 1 | SCIADUNA | | 202 | | 199 | 195 | 182 |
| 1 | SPINEDDU | | 202 | | 199 | 195 | 182 |
| 1 | UCCIARDUNI | | 202 | | 199 | 195 | 182 |
| 2 | ADAMO | | 202 | | 200 | 195 | 182 |
| 2 | ALESSIO | | 202 | | 200 | 195 | 182 |
| 2 | BELLA DI GIUGNO | | 202 | | 200 | 195 | 182 |
| 2 | BIANCHETTONE | | 202 | | 200 | 195 | 182 |
| 2 | BUONA LUISA | | 202 | | 200 | 195 | 182 |
| 2 | BUTIRRA | | 202 | | 200 | 195 | 182 |
| 2 | COSCIA | | 202 | | 200 | 195 | 182 |
| 2 | DECANA DEL COMIZIO | | 202 | | 200 | 195 | 182 |
| 2 | DR. GUYOT | | 202 | | 200 | 195 | 182 |
| 2 | DUCHESSA D'ANGIO' | | 202 | | 200 | 195 | 182 |
| 2 | GENTILE | | 202 | | 200 | 195 | 182 |
| 2 | HARROW SWEET | | 202 | | 200 | 195 | 182 |
| 2 | MAX RED BARTLETT | | 202 | | 200 | 195 | 182 |
| 2 | OLD HOME | | 202 | | 200 | 195 | 182 |
| 2 | P. Pyraster 10 | | 202 | | 200 | 195 | 182 |
| 2 | PERGOLESI | | 202 | | 200 | 195 | 182 |
| 2 | PERO ANGELICO | | 202 | | 200 | 195 | 182 |
| 2 | VILLALBA | | 202 | | 200 | 195 | 182 |
| 2 | PIRU MULINCIANO | | 202 | | 200 | 195 | 182 |
| 2 | PISCIAZZANU | | 202 | | 200 | 195 | 182 |
| 2 | PUTIRU D'ESTATE | | 202 | | 200 | 195 | 182 |
| 2 | RAZZUOLO ROSATA | | 202 | | 200 | 195 | 182 |
| 2 | S.CONO | | 202 | | 200 | 195 | 182 |
| 2 | TABACCARO | | 202 | | 200 | 195 | 182 |
| 2 | URZI' | | 202 | | 200 | 195 | 182 |
| 2 | VIRGOLESE | | 202 | | 200 | 195 | 182 |
| 2 | WILLIAM'S | | 202 | | 200 | 195 | 182 |
| 2 | ZIO PIETRO | | 202 | | 200 | 195 | 182 |
| 3 | ANGELICO DOPPIO | | 201 | | 199 | 195 | 182 |
| 3 | AZZONE DI CASSONE | | 201 | | 199 | 195 | 182 |
| 3 | BERGAMOTTO | | 201 | | 199 | 195 | 182 |
| 3 | BIANCHETTO | | 201 | | 199 | 195 | 182 |
| 3 | BIANCHETTO | | 201 | | 199 | 195 | 182 |
| 3 | IANCULIDDU | | 201 | | 199 | 195 | 182 |
| 3 | IAZZULEDDU | | 201 | | 199 | 195 | 182 |
| 3 | MEZZACAMPANA | | 201 | | 199 | 195 | 182 |
| 3 | MOSCATELLO NERO | | 201 | | 199 | 195 | 182 |
| 3 | PASQUALINO | | 201 | | 199 | 195 | 182 |
| 3 | PICCOLA DOLCE | | 201 | | 199 | 195 | 182 |
| 3 | PIRIDDA | | 201 | | 199 | 195 | 182 |
| 3 | S.GIOVANNI | | 201 | | 199 | 195 | 182 |
| 3 | ZUCCAREDDU | | 201 | | 199 | 195 | 182 |
| 4 | SPADONA | | 201 | | 199 | 195 | 183 |
| 4 | FACCIA DONNA | | 201 | | 199 | 195 | 183 |
| 4 | GAROFALO | | 201 | | 199 | 195 | 183 |
| 4 | IALUFARU | | 201 | | 199 | 195 | 183 |
| 4 | MOSCATELLO | | 201 | | 199 | 195 | 183 |
| 4 | S. GIOVANNINO | | 201 | | 199 | 195 | 183 |
| 5 | *P. amygdaliformis 7* | | 216 | | 199 | 195 | 182 |
| 5 | *P. Pyraster 1* | | 216 | | 199 | 195 | 182 |
| 5 | *P. Pyraster 5* | | 216 | | 199 | 195 | 182 |
| 6 | CAVALIERE | | 203 | | 199 | 195 | 182 |
| 6 | GARIBALDI | | 203 | | 199 | 195 | 182 |
| 7 | BUTIRRA HARDY | | 201 | | 199 | 195 | 184 |
| 8 | CATANESE | | 197 | | 199 | 195 | 182 |
